# Asialoglycoprotein receptor subunit *Asgr1a* loss results in attenuated cholesterol absorption in zebrafish fed a western diet

**DOI:** 10.1016/j.jbc.2026.113239

**Published:** 2026-06-09

**Authors:** Joshua T. Derrick, Tabea O.C. Moll, Darby W. Sweeney, Jeffrey Shin, Monika Svecla, Giuseppe Danilo Norata, Steven A. Farber

**Affiliations:** 1 Department of Biology, Johns Hopkins University, Baltimore, Maryland, USA; 2 Department of Pharmacological and Biomolecular Sciences, Università degli Studi di Milano, Milan, Italy

**Keywords:** apolipoprotein B, asgr1, cholesterol, liver, steatosis, triglycerides, zebrafish

## Abstract

One of the major pathways to clear glycoproteins from circulation is *via* the liver-specific asialoglycoprotein receptor. Loss of asialoglycoprotein receptor 1 (*ASGR1*), the major subunit of asialoglycoprotein receptor in humans, was found to correlate with lower levels of plasma apolipoprotein B-containing lipoproteins and a profound reduction in cardiovascular disease risk. However, the cell and molecular biology underlying this effect was unclear. Given that rodents carry their cholesterol largely in High Density Lipoprotein, we selected the zebrafish model to better understand the mechanism(s) of action of ASGR1. We first characterized all possible zebrafish *ASGR1* orthologs to identify zebrafish Asgr1 (*asgr1a*) from a collection of lectin-binding proteins. We then generated two independent mutations in *asgr1a* using CRISPR/Cas9. Neither mutation altered larval, juvenile, or adult B-lp numbers or sizes. However, when challenged with a Western Diet, *asgr1a* mutant zebrafish exhibited less hepatic steatosis and lower hepatic triglyceride levels compared to control animals. *Asgr1a* mutant animals also exhibited increased levels of fecal cholesterol, due to attenuated post-prandial absorption and upregulation of liver proteins known to be involved in B-lp metabolism (*e.g.,* microsomal triglyceride transfer protein, ApoA4). These data are consistent with the atheroprotective role of ASGR1 and reveal a previously unappreciated role for ASGR1 in modulating whole animal cholesterol flux.

The asialoglycoprotein receptor (ASGPR) is a well-known liver-specific receptor. Discovered in the 1980’s by Gilbert Ashwell and Anatol Morell, it is also referred to as the Ashwell receptor (1). The ASGPR was initially described to explicitly bind circulating glycoproteins without terminal-siayl groups (1, 2). After the removal of the siayl-groups, glycoproteins that expose terminal galactose or N-acetylgalactosamine (GalNAc) residues are bound by the ASGPR (3). Once bound by these ligands, the ASGPR undergoes clathrin-mediated endocytosis (4). Following dissociation of the ASGPR with its ligand, the receptor is returned to the cell surface, and the ligand undergoes degradation through the lysosomal pathway (5).

ASGPR is grouped into the larger class of C-type lectin proteins (CLECs), with its two subunits, ASGR1 and ASGR2, being the only two members of the CLEC4H family. Its closest relative is the CLEC10A receptor (for a recent review, see (6)). Both ASGR1 and ASGR2 contain a cytoplasmic domain with an endocytic motif, a transmembrane domain, followed by the extracellular carbohydrate recognition domain, and the C-type lectin domain (7). Though the two proteins are similar in sequence, ASGR1 is required for ASGPR function, while ASGR2 is dispensable (8, 9). Changes in ligand specificity can be achieved by different quaternary structures of the ASGPR subunits (10), specifically in the terminal Gal of GalNAc residues of the glycoprotein (7, 10, 11). These variations in glycan residues could allow for a wide range of potential ligands, including apoptotic liver cells (12), circulating tumor cells (13, 14), and viruses (15, 16, 17) such as SARS-CoV-2 (18), and lipoproteins (19, 20).

Lipoproteins are essential for transporting hydrophobic lipids and fat-soluble nutrients through circulation to peripheral tissues and are composed of a protein and phospholipid coat surrounding a neutral lipid core (21). Apolipoprotein B (APOB)-containing lipoproteins (B-lps) are produced by the intestine (these particles are known as chylomicrons) and the liver (these particles are known as Very-Low-Density Lipoproteins (VLDL)) to transport dietary and endogenous lipids to peripheral tissues (22, 23). APOB is a required, non-interchangeable scaffold protein for B-lps (24), that remains associated with the B-lp from its production in the ER (25) to lysosomal degradation after the uptake of the B-lp from circulation (26). Human APOB contains 28 possible glycosylation sites, 13 of which are known to be sialylated (27). Seven of these sites are predicted to be in the part of the APOB protein that binds to the Low-Density Lipoprotein (LDL) receptor (28). In circulation, lipases will bind B-lps and hydrolyze triglycerides and other lipids, effectively shrinking the size of B-lps, leading to the formation of chylomicron remnants or Low-Density Lipoproteins. Several overlapping mechanisms are responsible for the liver uptake of these degraded B-lps (20, 29, 30). The LDL receptor (LDLR), expressed in the liver, plays a prominent role in clearing small B-lps from circulation (30). However, since B-lps can remain in circulation for a prolonged time, they can become desialylated and thus become ligands for the ASGPR (20). In fact, in rabbits, desialylated B-lps are more rapidly internalized in smooth muscle cells, suggesting a potentially important role for the ASGPR in B-lp uptake (31).

Elevated levels of LDL are associated with an increased risk of cardiovascular disease (32). Various drugs, such as statins (33) and proprotein convertase subtilisin/kexin type 9 (PCSK9) inhibitors (34), work to increase cell-surface levels of LDLR to lower plasma LDL. In 2016, a genome-wide association study found that heterozygous loss of *ASGR1* in humans correlated with reduced plasma LDL levels and decreased CVD risk (35). Cell culture experiments mimicking the human 12 bp deletion showed that the mutation reduced protein levels of ASGR1 (35). Since the ASGPR is also known to bind and endocytose plasma LDL (20, 31), the association with reduced cardiovascular risk was paradoxical, given the fact that mutations in the LDL receptor that inhibit LDL uptake increase cardiovascular disease risk (36).

Recent work in HepG2 cells (37), mice (38, 39), and pigs (40) revealed that loss of *ASGR1* increases the levels of surface LDLR through both PCSK9-independent (37) and dependent (38, 39, 40) mechanisms. Data from cultured HepG2 cells suggest that ASGPR directly binds LDLR, facilitating LDLR degradation; consequently, loss of *ASGR1* leads to elevated amounts of LDLR on the cell surface (37). In mice, however, ASGR1 was found to release sterol regulatory element–binding proteins (SREBPs) from the ER (38). Thus, loss of the *Asgr1* gene led to ER-trapped SREBP, which in turn decreased levels of PCSK9 and increased surface LDLR numbers (38). In contrast, a separate mouse study indicated that loss of *Asgr1* does not affect LDLR but instead prevents the degradation of the liver X receptor (LXR) (39). The increased levels of LXR lead to the upregulation of the ATP-binding cassette (ABC) transporters *Abca1* and *Abcg5/g8*. ABCA1 facilitates reverse cholesterol transport to High Density Lipoproteins, while ABCG5/G8 shuttle cholesterol towards bile synthesis and fecal secretion; thus their upregulation results in reduced cholesterol in the liver (39, 40). Both studies reported that loss of *Asgr1* led to lower serum cholesterol and triglycerides, but observed contradictory effects on lipoproteins (38, 39). A third study in mice investigated double mutants of *Asgr1* and *Apoe* fed a Western Diet (WD), and also observed a reduction in circulating B-lps and cholesterol, reduction of atherosclerotic plaque, upregulation of circulating immune cells, and changes in liver lipid metabolism (41). In contrast to these more recent studies, no effect on lipoprotein levels was described in the first mutant mouse model of *Asgr1* (42). The differences noted between these different studies may have been due to the specific mutations of ASGPR in the different mouse models, suggesting that further comparative analyses are warranted.

While these studies were ongoing and given that there was no clear mechanism of action for ASGR1 emerging in the literature, we independently investigated the influence of *ASGR1* on lipoprotein metabolism in zebrafish. Zebrafish are a powerful, model organism to study lipoprotein metabolism (43, 44). Critical genes involved in mammalian lipoprotein metabolism have been identified in zebrafish (45, 46, 47, 48, 49, 50, 51). In contrast to mice, which primarily transport lipids through high-density lipoproteins (52), zebrafish show a similar lipoprotein profile to humans in that they transport a bulk of their lipids through B-lps and express the *cetp* gene (53), which allows transport of cholesterol esters to B-lps. Additionally, the availability of customizable diets (54, 55), and an *in vivo* reporter to quantify lipoproteins (56) make zebrafish a suitable model organism for studying lipoprotein metabolism (reviewed by (43, 44, 57, 58, 59, 60, 61, 62)).

Here, we identified the zebrafish ortholog for *ASGR1*, *asgr1a,* and generated two independent KO lines using CRISPR/Cas9. While larval zebrafish lacking ASGR1 showed no change in their lipoprotein profiles, adult zebrafish *asgr1a* mutants challenged with a WD developed less steatosis and stored less triglyceride in the liver while excreting more cholesterol in their feces compared to control animals. We use a fluorescent cholesterol analog, BODIPY-cholesterol, to show that this additional fecal cholesterol was due to reduced dietary cholesterol absorption. Adult livers also display changes in transcript and protein levels, including a greater than ten-fold increase in levels of microsomal triglyceride transfer protein (Mttp) and apolipoprotein A-IV (ApoA4). Together, our results highlight the importance of ASGR1 in regulating whole animal liver lipid metabolism and cholesterol flux.

## Results

### In silico analysis suggests complicated evolution of asgr1 in zebrafish

**Figure 1:**
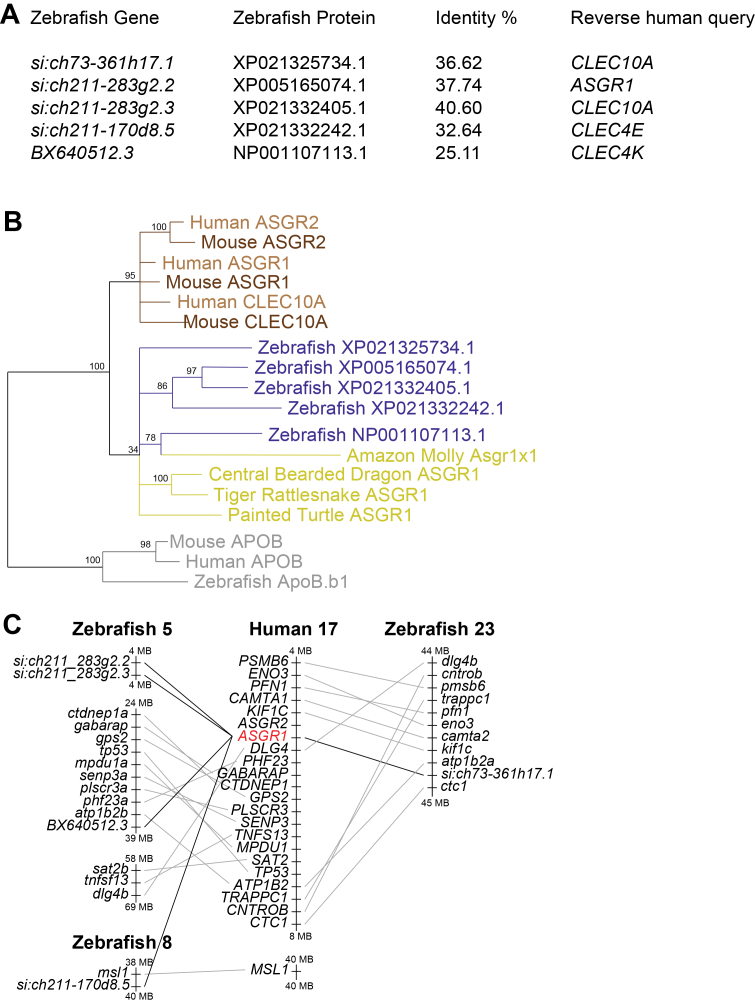
**Gene origin analysis for zebrafish asgr1 paralogs.***A*, BLAST of the human ASGR1 protein sequence to the zebrafish genome using standard settings on ensembl.org. *B*, phylogenetic tree based on amino acid sequences of human and mouse ASGR1, ASGR2, and CLEC10A to possible zebrafish orthologs and other non-mammalian ASGR1 orthologs. APOB sequence serves as an outgroup. *C*, syntenic analysis of human Chromosome 17 and possible zebrafish orthologs, not to scale. *Light gray lines* show synteny, black lines connect zebrafish asgr1 candidates.

While zebrafish share 70% of human coding genes (63), a BLASTp search of the zebrafish genome using human ASGR1 returned mostly unannotated genes (9 out of 20, Fig. 1*A*) and several genes of the C-type lectin family (6 out of 20), but no obvious ASGR orthologs. To identify the zebrafish ortholog for ASGR1, we narrowed down potential candidate genes using various criteria. Firstly, we selected the top three results of non-annotated genes, *si:ch73-361h17.1*, *si:ch211-283g2.2*, and *si:ch211-283g2.3,* which all showed similar identity scores (36.63–40.60%). Then, since human ASGR1 shares considerable similarities with ASGR2 and CLEC10A both in function and sequence (6), we also selected the unannotated gene *BX640512.3,* which was predicted to belong to the C-type lectin-binding protein family (25.11% identity with ASGR1). Finally, we included a recently described gene zebrafish hepatic lectin *si:ch211-170d8.5* (32.64% identity with ASGR1) (64). To determine which of these had the most similarity to human ASGR1, we used the pair-wise alignment of all zebrafish candidates using ClustalW, revealing that *si:ch73-361h17.1* had the highest overall similarity with human ASGR1 (42.4%) (Fig. S1).

We next turned to phylogenetic analysis based on amino acid sequences to further narrow down the five potential candidate genes. Human, mouse, and zebrafish APOB were chosen as an outgroup for performing a multiple alignment of the zebrafish ASGR1 sequences via MAFFT (https://mafft.cbrc.jp/alignment/software/) (65). In addition to ASGR1, we again included ASGR2 and CLEC10A from both the mouse and human in our alignment analysis, as well as ASGR1 proteins from reptile species as an additional outgroup (6). Based on the multiple alignments, MAFFT generated a neighbor-joining tree with bootstrap sampling. The mouse and human orthologs of ASGR1, ASGR2, and CLEC10A all form a clade with high bootstrap values, indicating that these mammalian orthologs share a common ancestor that probably diverged at some time after the evolutionary divergence between ray-finned and lobe-finned fishes (Fig. 1*B*).

All teleosts, including zebrafish, underwent a genome duplication event 350 mya (66, 67), thus, it is not uncommon for zebrafish to have more than one ortholog of a single human gene. Synteny analysis examines a gene of interest and its neighboring genes, as they tend to remain in proximity throughout evolution (68). Therefore, we compared the genes located near the various *asgr1* candidate genes in fish to neighboring genes of the ASGR1 locus in humans. *ASGR1* is located on human chromosome 17 at 7.17 MB. Syntenic analysis showed that the genes surrounding human *ASGR1* (4–40 MB examined) could be found in blocks on the zebrafish chromosomes 5 (*si:ch211-283g2.2*, *si:ch211-283g2.3*, and *BX640512.3)* and 23 (*si:ch73-361h17.1)*. The hepatic lectin candidate gene *si:ch211-170d8.5* is located on chromosome 8, but here there are only minor similarities of this chromosome to human chromosome 17 (Fig. 1*C*). In conclusion, we determined that the candidate *asgr1* genes likely lie on either chromosome 5 or 23, leaving us with four potential gene candidates. The paralogs on chromosomes 5 and 23 resulted from a genomic duplication of chromosome 5. The two additional candidate genes on chromosome 5 likely resulted from tandem duplication, which is also common in zebrafish (69).

### mRNA expression pattern of candidate zebrafish asgr1 paralogs

**Figure 2:**
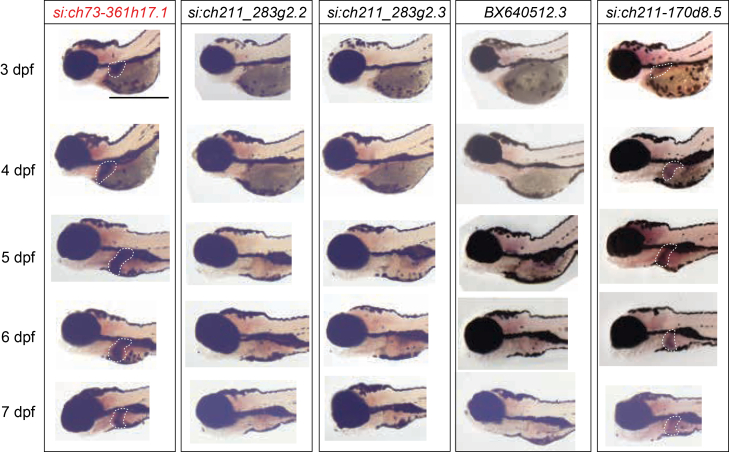
**mRNA expression of *asgr1* candidate genes during larval stages.** Whole-mount *in situ* hybridization for riboprobes of *si:ch73-361h17.1* and *si:ch211-170d8.5,* 3 to 7 dpf. The signal is confined to the developing liver (*white dotted outline*). For *si:ch211_283g2.2*, *si:ch211_283g2.3*, and BX640512.3 no signal was ever detected. Whole-mount *in situ* hybridization was performed three independent times per probe for all candidate orthologs at all developmental stages, n ≥ 6 in each experiment. *Si:ch73-361h17.1* showed liver signal consistent (36/36 fish) every experiment starting at 4 dpf and (29/31 fish) at 3 dpf. *Si:ch211-170d8.5* showed consistent signal (22/29 fish). Starting 4 dpf and (3/27 fish) at 3 dpf. The scale bar represents 1 mm.

In addition to sequence and syntenic analyses, the closest zebrafish ortholog to a human gene is often identified by examining the mRNA expression pattern with whole-mount *in situ* hybridization (WISH) using an antisense riboprobe (68). Determination of mRNA expression patterns is crucial when identifying zebrafish orthologs since the genome duplication after divergence from the vertebrate common ancestor often leads to silent copies of genes in zebrafish (66, 67, 70, 71, 72, 73, 74, 75). We performed WISH with specific anti-sense and sense riboprobes against all candidate mRNA sequences on whole zebrafish larvae. Our goal was to identify the candidate gene with liver-specific expression since *ASGR1* shows liver-specific expression in humans, while CLEC10A is ubiquitously expressed (6). Only the probes for *si:ch73-361h17.1* and *si:ch211-170d8.5* showed the liver expression. *Si:ch73-361h17.1* signal was detected in the liver at 3 dpf (n = 29 of n_total_ = 31), while only a subset of larvae showed *si:ch211-170d8.5* expression at 3 dpf (n = 3 of n_total_ = 27). At 5 dpf, both probes exhibited strong liver-specific mRNA expression (Fig. 2). Sense controls for all riboprobes showed no signal (Fig. S2).

These data support *si:ch73-361h17.1* being the *ASGR1* ortholog, given its higher similarity to human *ASGR1*, its syntenic position, and similar tissue expression profile. The correct nomenclature of these genes was determined in conjunction with the Zebrafish Information Network (ZFIN). The active gene is *si:ch73-361h17.1,* named *asgr1a,* and the non-expressing duplicate is *BX640512.3,* named *asgr1b.* The paralogs *si:ch211-283g2.2* and *si:ch211-283g2.3,* which do also not actively express mRNA, most likely evolved by tandem duplication and are named *asgr1c.1* and *asgr1c.2*, respectively. *Si:ch211-170d8.5* does show liver-specific expression; however, its distance in both phylogenetic and syntenic analysis supports its naming as *asgr-like* *1 (asgrl1)*. These genes and their locations are summarized in Table 1.

**Table 1:** ASGR1 orthologs and their locations

Gene location	Putative gene name
*si:ch73-361h17.1*	*asgr1a*
*BX640512.3*	*asgr1b*
*si:ch211-283g2.2*	*asgr1c.1*
*si:ch211-283g2.3*	*asgr1c.2*
*si:ch211-170d8.5*	*asgr-like1 (asgrl1)*

The gene locations (column 1) and putative gene names (column 2) of all identified zebrafish ASGR1 homologs.

### Generation of two asgr1a mutant zebrafish lines

**Figure 3:**
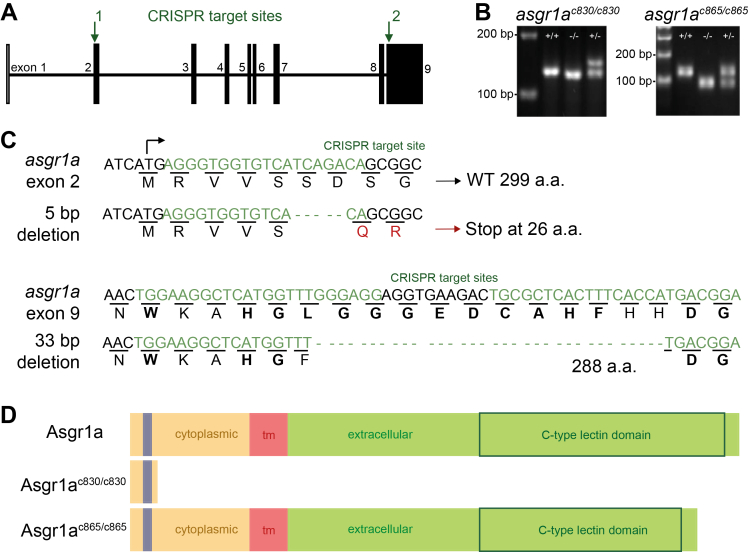
**Generating two mutations in *asgr1a* using CRISPR/Cas9.***A*, schematic of zebrafish *asgr1a* gene showing the CRISPR target sites in exon 2 and 9. *B*, representative images of genotyping assays for both mutations showing heteroduplex formation in *asgr1*^*830/+*^. *C*, sequence alterations in *asgr1a*^*c830/c830*^ (5 bp deletion in exon 2) and *asgr1a*^*c865/c865*^ (33 bp deletion in exon 9). Conserved amino acids to human ASGR1 are highlighted in *bold*. Red indicates the change in amino acid sequence, *green letters* denote the sequence of CRISPR target sites. *D*, predicted protein length and domains in WT and both *asgr1a* mutations. *Orange*: cytoplasmic region with the *blue* endocytosis motif, *red*: transmembrane domain, *green*; extracellular domain of the protein with a box highlighting the predicted area of the C-type lectin domain. Assignments were performed based on pairwise alignment of the amino acid sequence to human ASGR1 and its known functional domains.

Having identified the likely zebrafish *ASGR1* ortholog, we generated two independent *asgr1a* mutants using CRISPR/Cas9. For the first mutation, we targeted the start codon of *asgr1a* (Fig. 3*A*). This produced a 5-bp deletion in exon 2 (*asgr1a: c.15_20del*) with a predicted premature stop codon after 26 amino acids. This allele is now designated as allele c830 (*asgr1a*^*c830/c830*^) (Fig. 3, *B*–*D*). Sequencing of gDNA and cDNA (by RNA-seq analysis) of the mutated locus, confirmed the deletion (Fig. S3*A*). Heterozygous animals can be identified readily by heteroduplex formation on an agarose gel (76, 77). (Fig. 3*B*). We were concerned that alternative start sites could mask a phenotype since several potential start codons could lead to a partially functional protein (78, 79, 80). Furthermore, it is known that usually silent paralogs can compensate for reduced expression of an active gene (80, 81). To address these concerns, a second independent mutation in the predicted C-type lectin binding domain was generated. This domain is essential for binding glycoproteins; hence, its amino acid sequence shows more conservation than the other functional domains (Fig. S1*B*). By injecting three CRISPR guides spaced several bps apart, a 33 bp mutation was introduced in exon 9 (*asgr1a: c.791_824del;* allele c865). This mutation, *asgr1a*^*c865/c865*^, leads to the loss of nine conserved amino acids and changes one conserved amino acid (Figs. 3*C* and S3*B*), shortening the full-length protein to 288 amino acids compared to the wild-type sequence of 299 amino acids (Fig. 3, *B*–*D*). Homozygous mutants for both *asgr1a* mutations are viable, no visible phenotypes were noted in mutant embryos or larvae, and no changes in mass or length at 6 months of age were observed when mutants were fed a normal diet (Fig. S5, *A* and *B*). CRISPR and genotyping primers are listed in Table S1.

### asgr1a mutants do not exhibit changes in B-lp number or particle size

To measure the impact of *asgr1a* loss on B-lp metabolism in zebrafish, *asgr1a*^*c830/c830*^ and *asgr1a*^*c865/c865*^ were crossed into a B-lp reporter line called the LipoGlo (56). The LipoGlo reporter system allows for the quantification of B-lp numbers and estimation of B-lp sizes in individual zebrafish larvae through the fusion of Nanoluciferase (NanoLuc) to the endogenous C-terminus of Apobb.1, the predominant zebrafish ortholog of APOB (56, 82). Each B-lp contains a single copy of APOB as its structural scaffold (83), which is not interchangeable or separable from the particle. Therefore, the relative luminescence signal from the NanoLuc is proportional to the total amount of B-lps (56). We found that there was no detectable change in B-lp number or particle size in either *asgr1a* mutant when compared to their wild-type siblings during larval and juvenile stages (Fig. S4).

### asgr1a mutants are protected from WD-induced hepatic steatosis and excrete excess fecal cholesterol than wild-type siblings

Recent mouse studies, conducted both on WD and normal diets, reported that the loss of *Asgr1* led to less hepatic steatosis due to the secretion of excess dietary cholesterol in the feces (38, 39). To explore these phenotypes in our zebrafish *asgr1a* mutants, adult fish were challenged with a WD for 6 days; mutant *asgr1a*^*c830/c830*^, *asgr1a*^*c865/c865*^ fish, and wild-type controls were fed a WD (8% fructose, 7.5% soybean oil, 8% palm oil, 4% cholesterol, Sparos) twice a day. After the morning feed on the sixth day, fish were separated, and feces were collected before the fish were dissected to obtain both plasma and livers for analysis.

**Figure 4:**
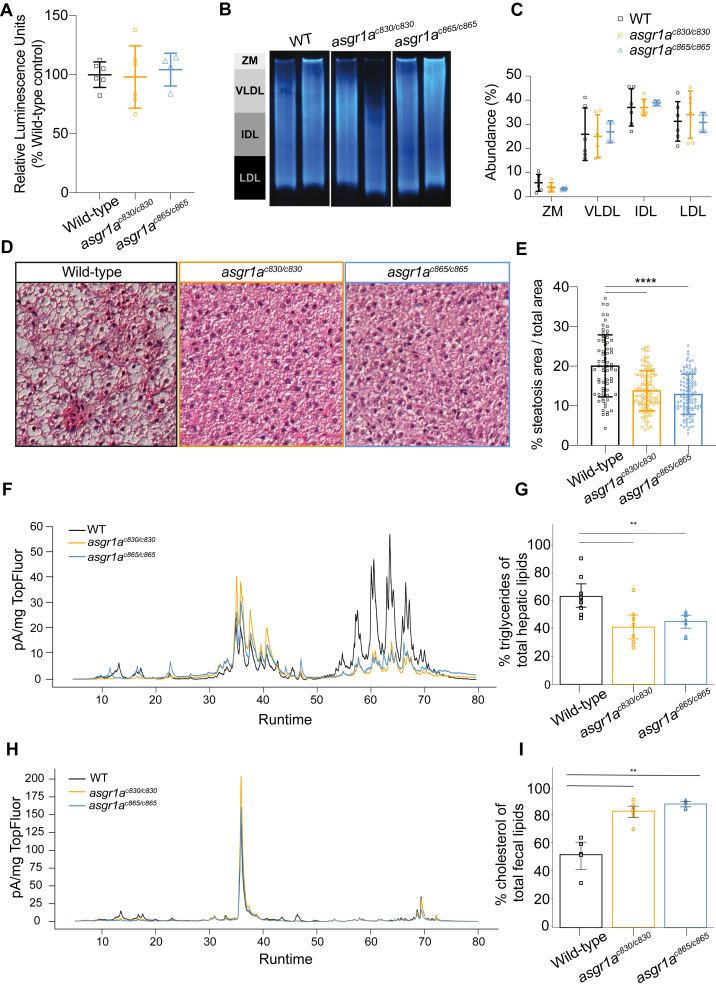
***asgr1a* mutants show less hepatic steatosis and lower levels of hepatic triglycerides but excrete more cholesterol in response to excess dietary cholesterol. Male zebrafish received a WD for 6 days before the blood, the liver, and the feces were collected.***A*, LipoGlo-Counting of plasma samples indicates B-lp quantity in *asgr1a* mutants and control animals. Two independent experiments, n = 2 to 3 fish each; two-way ANOVA followed by Tukey’s multiple comparison test. Experiments were normalized to their control group and shown in % to the control group. *B*, representative images of LipoGlo-Electrophoresis on plasma B-lps. Zero mobility and B-lp classes (VLDL, IDL, LDL) can be distinguished and analyzed. *C*, quantification of LipoGlo-Electrophoresis assay. Two independent experiments, n = 2 to 4 fish/genotype; two-way ANOVA followed by Tukey’s multiple comparison test. *D*, representative H&E images of liver steatosis. *E*, quantification of hepatic steatosis in *asgr1a*^*c830/c830*^ (13.82% ± 5.079%), *asgr1a*^*c865/c865*^ (12.90% ± 5.058%), and control animals (20.06% ± 7.829%). n = 9 from three independent experiments; two-way ANOVA followed by Tukey’s multiple comparison test. *F*, example HPLC trace of one sample of an *asgr1a* mutant *(blue and orange*) and control (*black*) livers. *G*, HPLC peak analysis of three independent experiments, n = 9, *asgr1a*^*c830/c830*^ (41% ± 14%) and *asgr1a*^*c865/c865*^ (45% ± 7.5%) mutants compared to controls (63% ± 13%). *H*, example HPLC trace of one sample of an *asgr1a* mutant (*blue and orange*) and control (*black*) fecal matter. *I*, peak analysis of three independent experiments, n = 9, of fecal cholesterol in *asgr1a*^*c830/c830*^ (83.6% ± 6.2%) and *asgr1a*^*c865/c865*^ (88% ± 2.6%) mutants compared to controls (52% ± 12.4%). Statistical analysis as follows: *E*, analyzed using two-way ANOVA followed by Tukey’s multiple comparison test. *G and I*, significance was computed using two Mann-Whitney *U*-tests with the Bonferroni correction. ∗∗ = *p* < 0.01; ∗∗∗∗ = *p* < 0.0001.

Mutant *asgr1a*^*c830/c830*^ and *asgr1a*^*c865/c865*^ fish on a WD did not exhibit changes in plasma B-lp levels or particle sizes compared to wild-type animals on a WD, as measured with the plasma LipoGlo-counting and electrophoresis assays (55) (Fig. 4, *A*–*C*). To examine the levels of liver steatosis, liver tissue was analyzed by H&E staining and HPLC (84). H&E analysis showed that there was a significant reduction in hepatic steatosis in males of both mutants for *asgr1a* compared to the controls in three independent experiments (Figs. 4, *D*, *E* and S6; WT: 20.06% ± 7.83; *asgr1a*^*c830/c830*^: 13.82% ± 5.08; *asgr1a*^*c865/c865:*^ 12.90% ± 5.06, *p* = 0.000001). HPLC analysis of the same liver samples revealed a decrease in triglycerides in both *asgr1a* mutants by about 20% (Fig. 4, *F* and *G* wild-type 60% ± 11; *asgr1a*^*c830/c830*^: 40% ± 9.7; *asgr1a*^*c865/c865:*^ 38% ± 9.3, *p* = 0.0079, 0.0037). Feces collected from the same fish and analyzed by HPLC revealed that *asgr1a* mutants excreted more cholesterol than the control animals (Fig. 4, *F* and *G* wild-type 43% ± 5.8; *asgr1a*^*c830/c830*^: 67% ± 17; *asgr1a*^*c865/c865:*^ 56% ± 7.4, *p* = 0.0016, 0.0043). Though we performed the same analyses in females, we found no phenotype in response to the WD feeding (Fig. S5, *C*–*G*), possibly due to the significant metabolic changes that occur with their 5-day hormonal cycle associated with egg laying (85, 86) (Fig. S5, *C*–*H*).

### WD induces changes in expression of metabolic processing genes and proteins in *asgr1a* mutants

**Figure 5:**
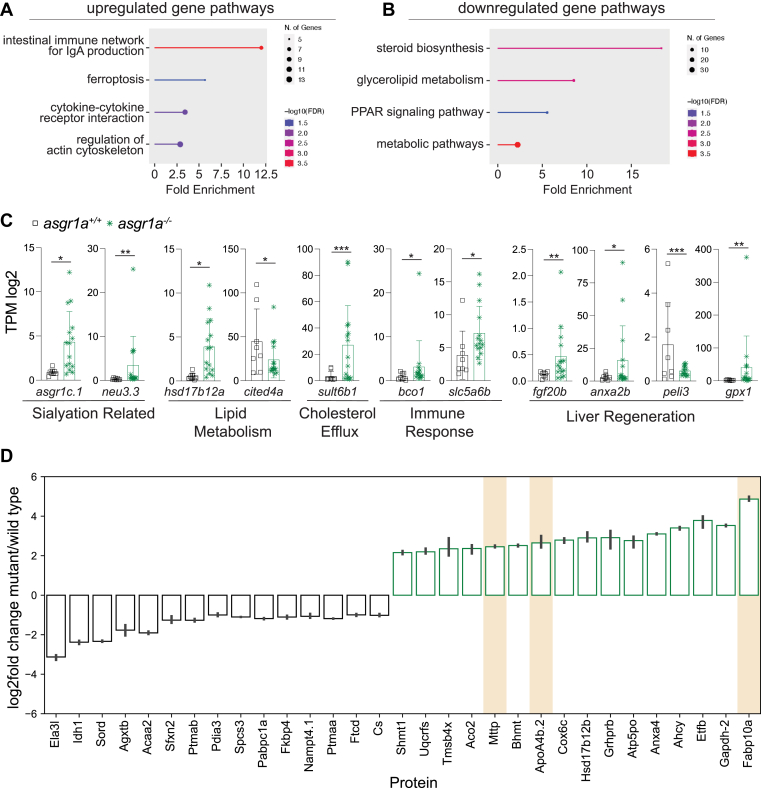
***asgr1a* mutants show differentially expressed cholesterol and lipid metabolism genes Male zebrafish received a WD for 6 days before livers were collected for RNA and protein analysis.***A*, Kegg pathway analysis of transcripts downregulated in combined mutant *asgr1a*^*c830/c830*^ and *asgr1a*^*c865/c865*^ vs WT livers. *B*, Kegg pathway analysis of transcripts upregulated in combined mutant *asgr1a*^*c830/c830*^ and *asgr1a*^*c865/c865*^*versus* WT livers. *C*, specific differentially regulated genes in sialyation, lipid-metabolism, cholesterol efflux, immune response, and the liver health-related pathways (N = 3, n = 6–8). Statistical significance was calculated using PyDeseq2, p-adjusted values, which correct for multiple comparisons, were used. ∗ = *p* < 0.05; ∗∗ = *p* < 0.01; ∗∗∗ = *p* < 0.001; ∗∗∗∗ = *p* < 0.0001). *D*, top 15 up- and downregulated proteins in mutant *asgr1a*^*c865/c865*^*versus* WT fish (n = 3 fish per genotype). Lipid-associated genes are highlighted in *gold*.

To investigate the mechanism by which *asgr1a* directs excess dietary cholesterol towards excretion instead of hepatic uptake, we performed RNAseq on the same livers analyzed by H & E and HPLC. While a KEGG Pathway analysis showed genes involved in immune pathways and ferroptosis are upregulated in liver (Fig. 5*A*), the most strongly downregulated pathways are steroid biosynthesis and metabolism (Fig. 5*B*), confirming that *asgr1a* is involved in metabolic regulation in zebrafish, similar to previously reported model organisms (38, 39). Individual genes described in previous studies, such as *ldlra* (1.23-fold decrease, *p* = 0.05, padj=0.58), *cyp7a1* (2.42-fold decrease *p* = 0.01, padj=0.37), and *cyp8b1.3* (2.7-fold decrease p = 0.006, padj=0.26), appear to be potentially downregulated, but are not significant when adjusted for multiple comparisons. This could be due to a 2.11-fold increase in transcript levels of *asgr1c.1* (padj = 0.017), a paralog for *asgr1a* we identified by syntenic and phylogenetic analysis (Fig. 1). Further, the transcriptomic analysis showed that the transmembrane sialidase (87) *neu3.3* is upregulated 3.19-fold in *asgr1a* mutants (*p* = 0.004).

The lower amounts of accumulated lipids in the liver are also supported by the 2.44-fold upregulation of *hsd17b12a* (*p* = 0.031), the 1.91-fold decrease of *cited4a* (*p* = 0.034), and the 3.17-fold increase of *sult6b1* (*p* = 0.0006). *Hsd17b12a* encodes a hydroxysteroid (17-beta) dehydrogenase, a key enzyme promoting long-chain fatty acid synthesis (88). Mammals lacking HSD17B12 have increased hepatic lipids due to the inability to form fatty acids longer than 18C (88). In mice, decreased levels of *Cited4* are correlated with less lipid droplet formation (89, 90) and as part of the sulfidation process, *sult6b1* may contribute to the hepatic secretion of cholesterol (41, 91).

While there is currently little evidence that zebrafish develop atherosclerotic plaques like mammals (92), some genes that are associated with lower atherogenic plaque burden in mammals are differentially regulated in our dataset. Specifically, we see a 2.25-fold increase (padj = 0.015) in *bco1*, which activates Treg cells and thereby promotes the clearance of plaques in mice (93). Further, *slc5a6b* is upregulated 1.86-fold (padj = 0.041) in *asgr1a* mutants, a gene that promotes B-lymphocytes maturation in mice (94).

Additionally, we found that the WD in *asgr1a* mutants leads to higher levels of several genes that could indicate improved liver health, such as *anxa2b* (2.01-fold increase, *p* = 0.043), *fgf20b* (2.56-fold increase, padj = 0.007), *peli3* (2.72-fold decrease, padj = 0.0002), and *gpx1* (3.43-fold increase, padj = 0.003). Specifically, the fibroblast growth factor, *Fgf20* (95), and the Glutathione peroxidase 1, GPx-1 (96), predict liver health in patient populations and lower levels of Pillion 3 protein, Pellino3, ameliorate liver injury (97). In zebrafish, the annexin gene *anxa2b* is upregulated during fin regeneration (98) and has been correlated in mammals with liver regeneration (99).

In addition to RNA-sequencing, we also performed proteomics on three *asgr1a*^*c865/c865*^ livers and three WT livers. We identified a total of 226 proteins (Table S2) that were present in both mutant and WT livers, which was too few to do a KEGG pathway analysis. We used an additional zebrafish liver proteomic dataset (100) to generate a more accurate background set of proteins and reran the KEGG analysis, obtaining hits in cytochrome P450 immunity, BCAA metabolism, and metabolic pathways (Fig. S7). More interestingly, when analyzing the top 15 up and downregulated genes, we observed a 100-fold up-regulation in expression of Fabp10a, a 14-fold increase in expression of ApoA4b.2, and 12-fold increase in expression of Mttp in mutant fish (Fig. 5*D*). Interestingly, none of these three genes has significantly different RNA-expression, although *apoa4b.2* was upregulated 2.4-fold (padj = 0.37). Mttp, ApoA4b.2, and Fabp10a are of special interest because of their known roles in liver lipid metabolism. Fabp10a (101) is a highly expressed a liver-specific fatty-acid binding protein, but has no mammalian homologs and has not been implicated in dyslipidemia. ApoA4b.2 is the most highly expressed form of ApoA4 in zebrafish (49), a protein that has been highly implicated in regulation of lipoprotein size (102, 103), lipoprotein clearance (104), and regulation of satiety (49, 105). ApoA4 has undergone duplication in teleosts, but the other copies are not differentialy expressed on a transcript or polypeptide level. Finally, Mttp is the zebrafish homolog of the mammalian MTP protein (82), which is essential for the synthesis of B-lps (22). We did not observe an increase in B-lp size or number, despite the upregulation of these proteins (Fig. 4*A*).

### *asgr1a*^*c830/c830*^ mutants absorb less BODIPY-cholesterol directly from a WD

**Figure 6:**
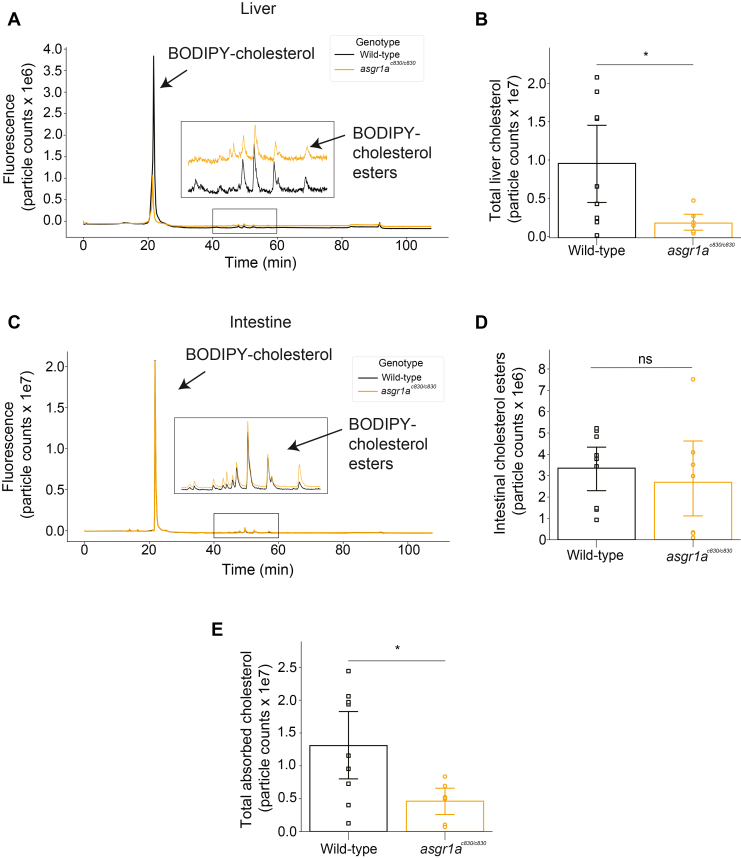
***asgr1a*^*c830/c830*^ mutants absorb less cholesterol after a high-cholesterol meal One-year-old male zebrafish fed a WD spiked with 1% BODIPY-cholesterol.***A*, example chromatograph of a WT (*black*) *versus asgr1a*^*c830/c830*^ (*orange*) liver. Free cholesterol and cholesteryl esters are labeled with *arrows*. *B*, quantification of combined BODIPY-cholesterol and cholesteryl ester peak areas of WT (9.6 e6 ± 4 e6 particles) and *asgr1a*^*c830/c830*^ (1.9 e6 ± 6 e5 particles) animals. *C*, example chromatograph of a WT (*black*) *versus asgr1a*^*c830/c830*^ (*orange*) intestine. *D*, quantification of intestinal BODIPY-cholesteryl ester peaks areas of WT (3.3 e6 ± 1.2 e6 particles) and *asgr1a*^*c830/c830*^ (2.6 e6 ± 1.6 e6 particles) animals. *E*, quantification of total absorbed cholesterol (the liver total cholesterol + intestinal cholesteryl ester) in WT (1.3 e7 ± 6.2 e6 particles) and *asgr1a*^*c830/c830*^ (4.6 e6 ± 2.8 e6) animals. Significance was computed using the Mann-Whitney U-test. ∗ = *p* < 0.05 (N = 2, n = 7–9).

To further elucidate the origin of increased fecal cholesterol levels of *asgr1a* mutants, we fed *asgr1a*^*c830/c830*^ and WT fish the WD spiked with BODIPY-cholesterol (106), a version of cholesterol tagged with the boron-based fluorophore BODIPY that behaves similarly to cholesterol (107). We chose two time points to dissect fish livers and intestines: 3 h and 2 days post feeding. The 3-h time point was intended to measure absorption: it is long enough after feeding for significant digestion and absorption to occur, while not long enough to allow for significant amounts of tracer to be incorporated into bile acids. To measure the quantity of free BODIPY-cholesterol and BODIPY cholesteryl esters, lipid extracts from the liver and intestinal tissue were subject to HPLC. The resulting chromatographs were divided into a region containing free-cholesterol or a region containing cholesteryl esters (Figs. 6, *A*, *C* and S8) (84) and quantified using PeakClimber (108). WT fish had approximately 5-times (9e6 vs 1.9e6 particles, *p* = 0.041) as much total cholesterol in their livers 3 h after feeding (Fig. 6*B*). Intestinal cholesteryl esters, a proxy for absorption because of the impossibility of distinguishing between free cholesterol in the intestinal lumen or enterocytes was also higher in WT animals, but this difference was not statistically significant (Fig. 6*D*). When combined, the total amount of absorbed cholesterol in the liver and intestine was nearly 3-times higher in WT animals (1.3e7 *versus* 4.6e6 particles, *p* = 0.0311), suggesting that dietary absorption of cholesterol is defective in *asgr1a*^*c830/c830*^ mutants (Fig. 6*E*). Differences in cholesterol secretion, either through the TICE pathway or bile acid synthesis, were also measured by dissecting livers fed the same dose of BODIPY-cholesterol 2 days after feeding. We did not observe a statistically significant difference in the amount of BODIPY-cholesterol in the liver 2 days post feeding (Fig. S9*B*), although there was slightly more BODIPY-cholesterol in WT animals, perhaps reflecting the increased uptake rate we observed 3 h post-prandially.

## Discussion

Concurrent with our study, five other independent groups started investigating the physiological response to loss of ASGR1 after it was reported to reduce LDL-cholesterol and the development of cardiovascular disease (35). Experiments in pigs and cultured cells indicated that lowered B-lps levels in *ASGR1* mutants were achieved *via* increased surface LDLR in hepatic cells (37, 40). In mice, two complete KOs of *Asgr1* were reported by two research groups. One mouse study indicated that ASGR1 acts on the LDLR through the SREBP pathway (38). The second mouse study reported a mechanism *via* LXR and did not see an effect on LDLR (39). While these two studies show contradicting results regarding total number and size profiles of B-lps, both reported an increase in fecal cholesterol in *Asgr1* mutants. A third mouse study analyzed *Apoe* and *Asgr1* double mutants, and also saw reductions in LDL and circulating cholesterol, activation of the immune system, as well as large changes in the liver proteome involved increases in fatty acid catabolism and production of bile acids (41). *Asgr1* and *Ldlr* double mutants in these mice had similar reductions in circulating cholesterol to single mutants.

We used a combination of phylogenetic and syntenic analysis, followed by *in situ* hybridization, to identify the zebrafish ortholog of *ASGR1*. These methods are often used together to help find functional, active zebrafish orthologs of human genes since zebrafish often have several orthologs of genes due to genome duplication and tandem duplication events (66, 67, 70, 71, 72, 73, 74, 75). While we identified the zebrafish orthologs for *ASGR1*, simultaneously, *asgr-like* *1* (64) and *asgr1a* (109) were described by other groups. In addition to expression pattern analysis by WISH and qPCR, both genes were reported to be involved in the immune response and clearance of pathogens from circulation (64, 109), which are known functions of the human ASGR1 (15, 16, 17, 18). We also see this in our own data: in zebrafish *asgr1a* mutants the most upregulated KEGG pathways involve the regulation of intestinal immunity (Fig. 5*A*).

We generated two independent mutations in *asgr1a* and did not see changes in lipoprotein numbers or particle sizes. This is in contrast to the GWAS findings that heterozygous loss of *ASGR1* in humans is correlated with reduced plasma LDL levels (35). As we could not detect any phenotype of *asgr1a* mutants under normal conditions (Figs. S4 and S5, *A*, *B*), we challenged zebrafish by feeding a WD. Indeed, when feeding the zebrafish a WD for 6 days, *asgr1a* mutants developed less steatosis and instead excreted the additional dietary lipids, especially cholesterol, in their feces, similar to some mouse phenotypes (38, 39). In these conditions, we still failed to see a change in circulating lipoproteins, despite a marked upregulation of both MTP and ApoA4 polypeptides in the livers of mutant animals. We also failed to observe many of the other transcriptional changes observed by other groups in expression of the LDL receptor (38) or bile pathways (39), but we did observe a downregulation of steroid biosynthesis and lipid metabolism in general, as well as changes in immunity which was observed by other groups (109). Part of this phenotype could be explained by compensation of the normally silent putative *asgr1c* gene identified in our orthology analyses. To fully replicate the phenotypes present in mice, it may be necessary to create a triple KO of *asgr1a*, *asgr1c.1* and *asgr1c.2*. However, even in mice, ASGR KOs displayed few changes in the circulating glycome, suggesting that there are potentially other C-type lectin receptors that can compensate for *Asgr1* loss (41).

However, even with compensation from *asgr1c*, we still observed protection from hepatic steatosis and increased levels of cholesterol in the feces on a WD. These two phenotypes are likely linked, as increased dietary cholesterol has been shown to drive liver and adipose fatty acid synthesis (55). High concentrations of cholesterol and cholesteryl esters in lipid droplets will result in the formation of crystals, which is damaging to the cell (110). Additional fatty acid synthesis protects hepatocytes from these crystals by reducing cholesterol concentrations in lipid droplets but causes steatosis. Reductions in body cholesterol levels *via* the feces is protective against this phenotype. An additional experiment with the fluorescent metabolic tracer BODIPY-cholesterol showed that the larger amounts of cholesterol in the feces is likely due to attenuated absorption in the intestine, as lower levels of BODIPY-cholesterol and its esterified products were present in the liver and intestine 3 h after a meal. This may be caused by increased levels of hepatic ApoA4, which may serve as a satiety signal that accelerates the transit time of the food bolus through the gut, reducing absorption. Further experiments are necessary to distinguish between these hypotheses and the LXR secretion model (39), as we also observed a mild, non-statistically significant decrease in stored BODIPY-cholesterol in the liver 2 days after a meal.

ASGPR is a receptor for many different particles, not just lipoproteins. Thus, part of the difficulty when establishing a mechanism of action for *asgr1a* on hepatic lipids is the polyvalent effect of ASGR1 on many different pathways. A recently published drug-target Mendelian randomization study examined the effects of genetically mimicked ASGR1 inhibitors. Consistent with our results that loss of ASGR1 affects a multitude of pathways, they found reduced levels of TG and APOB and also changes in insulin-like growth factor 1, albumin, and calcium (111). These effects are not surprising, given the variety of genes that we saw affected by the *asgr1a* mutation.

In summary, our results in zebrafish emphasize the importance of ASGR1 in modulating total fecal sterol levels and hepatic steatosis in the context of a WD, which may underlie human GWAS data indicating that attenuated ASGR1 function is cardioprotective. However, the mechanistic underpinnings that explain the relationship between ASGR1 function and fecal sterol levels still remain to be described.

## Experimental procedures

### Gene ancestry analysis

Sequence, syntenic, and phylogenetic analyses were performed to identify the asialoglycoprotein receptor 1 ortholog in zebrafish. All genetic and amino acid sequences were obtained from the National Center for Biotechnology Information (https://www.ncbi.nlm.nih.gov) and the Ensembl Genome Browser (ensembl.org).

Possible zebrafish orthologs were identified by querying the human protein sequence of *ASGR1*, transcript 201, on ensembl.org using BLASTp (standard settings: “Search Against”: Protein database “Proteins(Ensembl)”; “Search Sensitivities” set to “normal”, no Additional configurations) to the zebrafish RefSeq protein database (112, 113). The top three BLAST hits were recorded. A fourth candidate gene was chosen by looking at C-type lectin-containing protein families. The fifth candidate was a zebrafish hepatic lectin recently described by (59). Retro-BLASTp for all five potential zebrafish orthologs was performed. The amino acid sequences of Human, Mouse, Zebrafish, Central Bearded Dragon, Western Painted Turtle, Amazon Molly, and Tiger Rattlesnake for ASGR1, and the human and mouse orthologs for ASGR2 and CLEC10A were aligned using the iterative MAFFT multiple sequence alignment tool (65) to examine the phylogenetic evolution. For accurate guide tree building, APOB protein sequences for human, mouse, and zebrafish were chosen as outgroups (60). The G-INS-1 strategy was selected using standard settings (“Try to align gappy regions anyway”; Parameters: Scoring matrix for amino acid sequences: “BLOSUM62”; Gap opening penalty: “1.53”, Offset value: “0.0”; Score of N in nucleotide data: “(nzero) N has no effect on the alignment score”; Guide tree: “Default”; Mafft-homologs: “Use UniRef50 2019/Mar”), as it is recommended for accurate guide trees. Based on the alignment, the MAFFT tree server built a neighbor-joining tree with all gap-free sites, the JTT substitution model, and the estimation of site heterogeneity (alpha). Bootstrap resampling was set to 100, with bootstrap support ≥70 being interpreted as significant, and branches less than 50 were collapsed.

Optimal pairwise protein alignments between the human ASGR1 and all five potential zebrafish orthologs were determined with the ClustalW tool available in the MacVector (https://macvector.com) software. BLOSUM was selected while other settings were kept standard. Identity and similarity scores for human ASGR1, ASGR2, and CLEC10A to all five potential orthologs were determined with multiple alignments, the same settings as previously noted.

The syntenic relationship between the human ASGR1 and possible zebrafish gene loci was established using Ensembl Genome Browser by manually searching for zebrafish orthologs of genes located on human Chromosome 17, 4 to 40 MB (ASGR1 located at 7,173,431–7,179,370 MB).

### Zebrafish husbandry

AB (ZFIN ID: ZDB-GENO-960809-7) animals that were used for mutant generation were obtained from ZIRC ApoB-NanoLuc (56) animals (ZFIN ID:ZDB-FISH-200505-1) were used as lipoprotein reporter lines in this study.

Embryos were collected from the natural spawning of group crosses with two females and two males. Embryos were staged (114) and kept in embryo medium at 28 °C on a 14:10 light: dark cycle until 6 days post fertilization (dpf). Larvae were then moved to the fish facility (14:10 light: dark cycle) and fed with GEMMA Micro 75 (Skretting) three times a day until 14 dpf, GEMMA Micro 150 three times a day, and Artemia once daily from 15 to 42 dpf. Adult zebrafish were fed once daily with ∼3.5% body weight Gemma Micro 500 (Skretting). Fish challenged with a WD were fed similarly, but with WD, composed of the normal Sparos fish food plus 8% fructose, 7.5% soybean oil, 8% palm oil, and 4% cholesterol, twice a day. Fish were fed with this WD for 6 days before downstream experiments.

The Carnegie Institution Department of Embryology Animal Care and Use Committee (Protocol #139) approved all animal experiments.

### WISH

WISH was performed as previously described (115) to identify the expression of ortholog candidate genes for *asgr1* in zebrafish. Unique cDNA sequences for *asgr1a*, *asgr1b*, *asgr1c.1*, and *asgr1c.2* were synthesized by Genewiz (sequences listed in Table S1). The riboprobe for *asgrl1* was generated from cDNA according to (64). All riboprobes were cloned using TOPO cloning into pCRII (Thermo Fisher Scientific, K461020). Translation with Sp6 and T7 (Millipore Sigma, 10810274001 and 10881767001) generated sense and antisense digoxigenin-labeled riboprobes (Anti-Digoxigenin-AP, Fab fragments, Roche 11093274910). WISH was performed three times with an n > 5 on AB larvae stages 3 to 7 dpf and on 6 dpf larvae carrying either the *asgr1a*^*c830/c830*^ or the *asgr1a*^*c865/c865*^ mutation. Fish were imaged using a Nikon SMZ1500 microscope with HR Plan Apo 1× WD 54 objective, Infinity 3 Lumenera camera and Infinity Analyze 6.5 (https://www.teledynevisionsolutions.com/products/infinity-analyze/?model=infinityanalyze&vertical=tvs-lumenera&segment=tvs) software.

### CRISPR/Cas9 mediated KO

CRISPR target sites were identified with the help of the UCSC Genome Bioinformatics tool (https://genome.ucsc.edu/). The zebrafish genome was chosen, the locus for *asgr1a* was entered, and “CRISPR” under “Genes and Predictions” was set to “show”. Potential PAM sites were selected, and by clicking on the site, the guide sequence was displayed. Areas with high efficiency were chosen and ordered from Eurofins Genomics (supplementary primers 7–10). Oligos were resuspended into a 10 μM working stock and set up in a PCR reaction to prime to the CRISPR tail (primer 13) with dNTPs and high-fidelity polymerase (Phusion polymerase, Thermo Fisher Scientific #F-530S) (T_a_ = 55 °C, extension time 30 s, 15 cycles). After purification using the Zymo DNA Clean and Concentrate kit (Zymo Research, D4013), *in vitro* transcriptions into sgRNA were performed using the MEGAshortscript T7 kit (Thermo Fisher Scientific, AM1354). The sgRNAs were cleaned using the Zymo RNA clean and concentrate (Zymo Research, R1013). AB zebrafish were injected with a 2 nl injection mix (1500 ng/μl) at the 1-cell stage and raised to adulthood for founder identification. Founders were identified by out-crossing to WT AB fish and genotyping their offspring through PCR and sequencing. Primers used to generate guides are listed in Table S1.

### DNA extraction and genotyping

Genomic DNA was obtained by adapting the HotSHOT DNA extraction protocol (89). Adult fin clips were heated to 95 °C for 20 min in 50 μl of 50 mM NaOH, single larva in 20 μl. After cooling to room temperature, 1/10th volume of 1 M Tris pH 8.0 was added.

Genotyping primers for both *asgr1a* mutants were designed using ApE and synthesized by Eurofins Genomics. The locus around the 5 bp mutation in exon 2 of *asgr1a* was amplified using primers 1 and 2 at 0.5 μM concentration (T_a_ = 56 °C, extension time 20 s, 36 cycles). The WT band is 130 bp, and the homozygous mutant band is 125 bp. Heterozygous mutants show heteroduplex behavior in gel electrophoresis where bands run at approximately 125 bp and 140 bp. The locus of the 33 bp mutation in exon 9 of *asgr1a* was amplified using primers 3 and 4 (0.5 μM primer, T_a_ = 52 °C, extension time 40 s, 34 cycles). The WT band is 129 bp, and the homozygous mutant band is 96 bp. Primer sequences used for genotyping are listed in Table S1. For the Apobb.1-NanoLuc genotyping protocol, see (56).

### LipoGlo assays

See (56) for detailed LipoGlo methods. All reagents were obtained from Promega Corp., (N1110; (36)). Larval, juvenile, and adult *asgr1a*^*c830*^ mutants, as well as adult *asgr1a*^*c865*^ mutants carried one copy of the LipoGlo reporter (*apobb.1*^*Nluc/+*^), while larval experiments with *asgr1a*^*c865*^ animals carried two copies (*apobb.1*^*Nluc/Nluc*^).

To measure circulating lipoprotein levels in adults, blood was collected at the time of dissection by cutting off the tail fin and squeezing the body to collect the blood using EDTA-coated Kunststoff Kapillaren tubes (Sanguis Counting). The blood was centrifuged in a 1.5 ml Eppendorf tube for 2 min at 5000 g at 4 °C, and the plasma (1–2 μl) was pipetted into a new 1.5 ml Eppendorf tube and snap-frozen on dry ice. For larval LipoGlo assays, larvae were dispensed into 96-well plates (USA Scientific, #1402-9589) for homogenization in a total volume of 100 μl of 2x B-lp stabilization buffer (40 mM EGTA, pH 8.0, 20% sucrose + cOmplete Mini, EDTA-free protease inhibitor (Sigma, 11836170001)). Juveniles were dispensed into 96-well opaque white OptiPlate (PerkinElmer, 6005290) in a total volume of 200 μl of B-lp stabilization buffer. Homogenate was generated by sonication in a microplate-horn sonicator (Qsonica Q700 sonicator with a Misonix CL-334 microplate horn assembly) and kept on ice for immediate use before being stored at −20 °C. LipoGlo-Counting levels were obtained by mixing 1 μl of frozen plasma with 99 μl of diluted 1× B-lp stabilization buffer, or by mixing 40 μl of larval or juvenile homogenate with 40 μl of diluted LipoGlo buffer (1:3 NanoGlo buffer: PBS + 0.5% NanoLuc substrate) in a 96-well opaque black OptiPlate (PerkinElmer, 6005270).

All plates were read within 5 min of buffer addition. The plate for *asgr1a*^*c830/c830*^ was read on a SpectraMax M5 plate reader (Molecular Devices) set to top-read chemiluminescent detection with a 500 ms integration time. *Asgr1a*^*c865*^ LipoGlo-Counting plates and adult plasma LipoGlo-Counting samples were read on the BioTek Synergy H1 (Agilent) set to Luminescence Endpoint, 500 ms integration time.

LipoGlo-Electrophoresis was performed according to (56). Native-PAGE was imaged using the Odyssey Fc (LI-COR Biosciences) gel imaging system. For quantification, gels were analyzed using FIJI. Each lane was converted to a vertical plot profile. The Di-I LDL standard migration assigned areas for Zero Mobility, VLDL, IDL, and LDL.

### Tissue histology

Adult male zebrafish (12 months age-matched for all genotypes) were fed a WD (see zebrafish husbandry) for 6 days. Fish were euthanized by high-dose exposure to tricaine (Sigma-Aldrich, A5040-25). Through ventral sections, livers were removed as intact as possible and fixed in neutral-buffered formalin (Sigma, F8775) at RT for 24 h. The Johns Hopkins University Oncology Tissue Services performed sectioning and H & E staining. Slides were imaged with a Nikon E800 microscope with 20×/1.4 Plan Apo Nikon objective and Canon EOS T3 camera using EOS Utility image (https://app.ssw.imaging-saas.canon/app/en/eu.html) acquisition software. Quantification of steatosis was performed using FIJI (https://imagej.net/software/fiji/). Images were converted to RGB stacks, the threshold was set to 130 to 255, and the percentage area with steatosis was determined.

### HPLC

The quantification of lipid classes was performed by using HPLC with a charged aerosol detector. Tissues and fecal matter were suspended in 500 μl of 20 mM tris, 1 mM EDTA pH 7.8 lipid extraction buffer, and homogenized using a Thermo Fisher Scientific 550 Sonic Dismembrator. Using the Bligh-Dyer method (116), lipids were extracted from individual tissue homogenates and feces, then dried to 1% volume and resuspended in HPLC-grade isopropanol (>99.9% pure). The HPLC system (Thermo Fisher Scientific) with a C18 column and a CAD was used to analyze the lipid components of each sample. A gradient mobile phase was used for separating different lipid classes: 0 to 5 min = 0.8 ml/min in 98% mobile phase A (methanol-water-acetic acid, 750:250:4) and 2% mobile phase B (acetonitrile-acetic acid, 1000:4); 5 to 35 min = 0.8 to 1.0 ml/min, 98 - 30% A, 2 to 65% B, and 0 to 5% mobile phase C (2-propanol); 35 to 45 min = 1.0 ml/min, 30 - 0% A, 65 to 95% B, and 5% C; 45 to 73 min = 1.0 ml/min, 95 - 60% B and 5 to 40% C; and 73 to 80 min = 1.0 ml/min, 60% B, and 40% C. The amount of lipid in each sample was normalized to both the dry-weight of the samples and an internal lipid extraction control of TopFluor Cholesterol that was spiked into the lipid extraction solution and read on the fluorescence channel. For the figures generated in this paper, lipid classes were separated by runtime as established by (84), with fatty acids running from 5 to 30 min, phospholipids running from 30 to 45 min, and triglycerides and cholesteryl esters running from 45 to 75 min. Free cholesterol runs at 35 min. To calculate the quantity of each of these lipid classes, PeakClimber (108) was used to calculate individual peak areas. Peaks were then summed together in regions of interest and divided by the sum of all peaks in a trace to calculate the fraction of total area.

### RNA-seq of liver tissue

Liver tissues were collected as described earlier and snap-frozen on dry ice until further processing. The NucleoSpin RNA extraction kit (Macherey-Nagel, 740955.50) was used for RNA extraction. RNA-Seq was performed with the PolyA selection method using the TruSeq Stranded mRNA Library Prep Kit (Illumina, 20020595) with TruSeq single Indexes (Illumina, #20020492 and #20020493). The libraries were run on the NextSeq 500 (Illumina) with a 75 bp length single-end read at 50 M reads/sample.

Sequencing data was processed using the nf-core/rnaseq/3.12.0 pipeline (117), GRCz11 genome, and Ensembl Release 110 annotation. Statistical analysis of the sequencing data was performed using the PyDeseq2 library (118). Pathway analysis was performed using DAVID (119). All genes that were up- or downregulated over 1.5-fold, regardless of significance were included in the analysis.

### Liver proteomics and in-solution tryptic digestion

Livers were dissected and flash frozen before protein extraction. Liver protein extraction was performed as in (120). Liver tissue from control and ASGR1-deficient zebrafish (n = 3/group) was homogenized on ice in 8 M urea, 0.1 M Tris-HCl (pH 8.5) supplemented with protease inhibitors (1:100, Cell Signaling) as previously described (41). Briefly, after 60 min of shaking at 4 °C, the homogenates were centrifuged at 14,000×*g*, 30 min, 4 °C. Protein concentration in the supernatant was determined with the Lowry assay, and 10 μg of protein was dried in a vacuum concentrator for 45 min. The resulting pellet was resuspended in 10 μl water plus 10 μl 50 mM ammonium bicarbonate (final pH 8.5), reduced with 5 mM DTT (55 °C, 30 min), and alkylated with 150 mM iodoacetamide at room temperature for 30 min in the dark. Proteins were digested overnight at 37 °C with sequencing-grade trypsin (enzyme-to-protein ratio 1:20, w/w), and the reaction was stopped by adding 1% trifluoroacetic acid.

### LC-MS/MS analysis and proteomics data processing

LC-MS/MS and proteomics data processing was performed as in (120). Samples were acquired in Dionex Ultimate 3000 nano-LC system connected to an orbitrap Fusion Tribrid Mass Spectrometer (Thermo Fisher Scientific) equipped with a nanoelectrospray ion source. The peptide mixtures were pre-concentrated onto an Acclaim PepMap C18 5 μm, 100 Å, 100 μm ID x 2 cm (Thermo Fisher Scientific) and separated at 35 °C on an EASY-Spray PepMap RSLC C18 column: 3 μm, 100 Å, 75 μm ID × 25 cm (Thermo Fisher Scientific), using mobile phase A (0.1% aqueous formic acid) and mobile phase B (0.1% aqueous formic acid/acetonitrile (2:8)) at a flow rate: 300 nl/min. MS spectra were collected for the *m/z* ranges of 375 to 1500 Da, at 120,000 resolution, in the data dependent mode, cycle time 3 s between master scans, operating in positive ion mode. Fragmentation was induced by higher energy collisional dissociation with collision energy set at 35 eV. MS raw data files were converted to mzML format (centroid mode) using the MSconvert tool of the software ProteoWizard (https://proteowizard.sourceforge.io) (ver. 3.0.1957) (121).

Data were run through the Spectrum Recalibration and Precursor Detector nodes then searched against the Uniprot *Danio rerio* database (Downloaded 7/1/24) using Sequest HT within Proteome Discoverer (122) version 3.1 in a match between runs format for the total sample set. False discovery rates were calculated with the Target Decoy Peptide-Spectrum Match Validator node and data were filtered at 1% FDR. Quantification was performed with the Minora Feature Detector processing node followed by the Feature Mapper and Precursor Ions Quantifier nodes which aligned all chromatographic peaks of the same mass to charge ratio and calculated values based on precursor intensities. The intensities of the three replicates were normalized to Total Peptide Amount and the median normalized abundance of the group was used to calculate proteins as Summed Abundances of the connected peptide groups. The final protein abundance ratio was calculated as the normal space value of the Mutant over Control. KEGG analysis was performed by using the total liver proteome dataset from (100) combined with our identified proteins as a background group and taking all peptides with ± 1 log2-fold change and *p*-values of less than 0.05 as the differentially expressed group.

### Fluorescent cholesterol feeding

One-year old adult fish were challenged with the WD for 6 days as described previously. On the seventh day, fish were fed the same WD but spiked with 1% BODIPY-cholesterol (Cayman Chemicals: 24618). Fish were fed in individual tanks to ensure equal feeding and livers and intestines were dissected either 3 h or 2 days after feeding. Lipid extractions and HPLC were performed as described previously. Fluorescence readings were taken at excitation/emission of 488/520 nm with a sensitivity of 5 for the first 25 min and a sensitivity of 8 for the remainder of the run. Free cholesterol ran at 21 min, earlier than unlabeled cholesterol due to the charged fluorophore, and cholesteryl esters were quantified between 40 and 60 min.

### Statistics

GraphPad Prism (GraphPad Software, https://www.graphpad.com/features), or the Python packages seaborn and statannot, were used for graphing and statistical analysis. Outliers in all datasets were identified by ROUT and excluded from the analysis. Ordinary two-way ANOVA with Tukey’s multiple comparison test was performed on all developmental time courses of both *asgr1a* mutant LipoGlo experiments and the H&E quantification. Figure legends include sample sizes and details of statistical analysis.

## Data availability

All data used to generate figures in the manuscript is included in the supplemental “raw data quantification” excel file. Additional raw data, such as trace files of chromatographs is available at https://github.com/ATiredVegan/ASGR1a_2026_Derrick_Moll-Data-repository. Raw reads for RNA-seq can be accessed at NCBI GEO with the accession number GSE330025. Raw protein reads are available *via* PRIDE with accession number PXD078307.

## Supporting information

This article contains supporting information.

## Conflict of interest

The authors declare that they have no conflicts of interest with the contents of this article.
